# Common variants in the *ARC* gene are not associated with cognitive abilities

**DOI:** 10.1002/brb3.376

**Published:** 2015-09-03

**Authors:** Craig Myrum, Sudheer Giddaluru, Kaya Jacobsen, Thomas Espeseth, Lars Nyberg, Astri J. Lundervold, Jan Haavik, Lars‐Göran Nilsson, Ivar Reinvang, Vidar M. Steen, Stefan Johansson, Karin Wibrand, Stephanie Le Hellard, Clive R. Bramham

**Affiliations:** ^1^ Dr. Einar Martens Research Group for Biological PsychiatryCenter for Medical Genetics and Molecular MedicineHaukeland University HospitalBergenNorway; ^2^ Department of BiomedicineUniversity of BergenBergenNorway; ^3^ K.G. Jebsen Center for Research on Neuropsychiatric DisordersUniversity of BergenBergenNorway; ^4^ K.G. Jebsen Center for Psychosis Research and the Norwegian Center for Mental Disorders Research (NORMENT)Department of Clinical ScienceUniversity of BergenBergenNorway; ^5^ Department of PsychologyUniversity of OsloOsloNorway; ^6^ Norwegian Center for Mental Disorders Research (NORMENT) and the KG Jebsen Center for Psychosis ResearchDivision of Mental Health and AddictionOslo University HospitalOsloNorway; ^7^ Aging Research CenterKarolinska InstitutetStockholmSweden; ^8^ Umeå Center for Functional Brain ImagingUmeå UniversityUmeåSweden; ^9^ Department of Biological and Medical PsychologyUniversity of BergenBergenNorway; ^10^ Department of Clinical ScienceUniversity of BergenBergenNorway

**Keywords:** Activity‐regulated cytoskeleton‐associated protein, cognition, genetics, memory, single‐nucleotide polymorphism, synaptic plasticity

## Abstract

**Introduction:**

The Activity‐Regulated Cytoskeleton‐associated (*ARC*) gene encodes a protein that is critical for the consolidation of synaptic plasticity and long‐term memory formation. Given *ARC*'s key role in synaptic plasticity, we hypothesized that genetic variations in *ARC* may contribute to interindividual variability in human cognitive abilities or to attention‐deficit hyperactivity disorder (ADHD) susceptibility, where cognitive impairment often accompanies the disorder.

**Methods:**

We tested whether *ARC* variants are associated with six measures of cognitive functioning in 670 healthy subjects in the Norwegian Cognitive NeuroGenetics (NCNG) by extracting data from its Genome‐Wide Association Study (GWAS). In addition, the Swedish Betula sample of 1800 healthy subjects who underwent similar cognitive testing was also tested for association with 19 tag SNPs.

**Results:**

No *ARC* variants show association at the study‐wide level, but several markers show a trend toward association with human cognitive functions. We also tested for association between *ARC* SNPs and ADHD in a Norwegian sample of cases and controls, but found no significant associations.

**Conclusion:**

This study suggests that common genetic variants located in *ARC* do not account for variance in human cognitive abilities, though small effects cannot be ruled out.

## Introduction

The immediate early gene, *ARC/ARG3.1* (activity‐regulated cytoskeleton‐associated protein/activity‐regulated gene 3.1; here denoted as “*ARC*” for the gene and “Arc” for the mRNA and protein), controls diverse forms of experience‐dependent synaptic plasticity and memory formation in the mammalian brain (Plath et al. 2006; Bramham et al. 2010; Korb and Finkbeiner 2011; Shepherd and Bear 2011). *ARC* is expressed predominantly in excitatory, glutamatergic projection neurons suggesting late evolutionary emergence and functional specialization (Campillos et al. 2006; Vazdarjanova et al. 2006; Mattaliano et al. 2007). Following bursts of synaptic activity, Arc mRNA is rapidly induced and transported to dendritic processes for local storage, translation, or decay. Synthesis of Arc is required for long‐term potentiation (LTP) and long‐term depression (LTD), the major cellular mechanisms for synaptic strengthening and weakening, respectively (Chowdhury et al. 2006; Plath et al. 2006; Rial Verde et al. 2006; Shepherd et al. 2006; Messaoudi et al. 2007; Panja et al. 2014). Recent studies show that the Arc protein self‐oligomerizes, contains two biochemically distinct domains that flank a disordered region, and originated from the Ty3/Gypsy retrotransposon family (Myrum et al. 2015; Zhang et al. 2015).

Dysregulation of excitatory synaptic transmission and plasticity is increasingly implicated in the major psychiatric disorders, including bipolar disorder (BP), schizophrenia (SCZ), major depressive disorder (MDD), attention‐deficit hyperactivity disorder (ADHD), and autism spectrum disorders (ASD) (Toro et al. 2010; Auerbach et al. 2011; Grant 2012; Gkogkas et al. 2013; Nithianantharajah et al. 2013; Ripke et al. 2013; The Network and Pathway Analysis Subgroup of the Psyciatric Genomics Consortium 2015). Synaptic dysfunction may therefore play a causal role in cognitive impairments, such as in inhibitory control and other aspects of executive function, that are shared across all of the major neuropsychiatric disorders (Kahn and Keefe 2013). Numerous genetic risk factors are implicated in psychiatric disorder susceptibility (Sullivan et al. 2012). SCZ and BP heritability is estimated to be as high as 70%, whereas ADHD heritability is estimated at 79% (Lichtenstein et al. 2009, 2010). For SCZ, common genetic variants identified by Genome‐Wide Association Studies (GWAS) currently account for up to 18% of this heritability (Visscher et al. 2012; Ripke et al. 2014), but the heritability explained is lower for other psychiatric traits. Studies looking at the overall effect of common variants (present in more than 1% of the population) have shown that common variants could actually explain 40% of the heritability (Lee et al. 2012), but we are lacking either the sample size or the statistical tools to identify these common variants of small effect. Thus, many common variants of small effect in psychiatric disorders remain undetected. One approach to increase the power to identify these variants is to look at additional phenotypes which are correlated and relevant to these psychiatric traits, such as cognitive abilities (Fernandes et al. 2013; McIntosh et al. 2013; Lencz et al. 2014). As a functionally versatile regulator of plasticity and memory formation, the *ARC* gene, mRNA, and protein are all tightly regulated (Bramham et al. 2010). Dysregulation of Arc is implicated in Angelman syndrome and pathogenesis of Alzheimer's disease (Greer et al. 2010; Wu et al. 2011).

We hypothesized that genetic variations in *ARC* also could contribute to the range of human cognitive abilities and impairments in cognition. Previously, postsynaptic proteins at glutamatergic synapses associated with Arc and N‐methyl‐D‐aspartate receptor complexes have been shown to be enriched in SCZ‐associated loci containing copy number variants (CNVs), rare coding variants, and small de novo mutants (Kirov et al. 2012; Fromer et al. 2014; Purcell et al. 2014) Here, we carried out a comprehensive association analysis of *ARC* genetic variants to determine whether *ARC* variation plays a role in general cognition. First, we tested genetic variants in *ARC* for their association with cognitive abilities (word comprehension, visuospatial ability, intellectual function, verbal learning, verbal recall, and response inhibition) in two samples with cognitive phenotyping: the Norwegian Cognitive NeuroGenetics sample (NCNG) (Espeseth et al. 2012) and the Betula sample (Nilsson et al. 1997, 2004). Furthermore, we checked for association between *ARC* SNPs and a Norwegian ADHD sample (Jacobsen et al. 2013) since ADHD patients present with deficits in cognitive abilities, particularly in executive function (Pennington and Ozonoff 1996).

## Materials and Methods

The study utilized three independent datasets, which are described here. A summary of these cohorts are summarized in Table 1.

**Table 1:** Summary of cohorts

Cohort	Sample size	Mean Age ± SD
NCNG	645	47.6 ± 18.26
Betula	1742	62.3 ± 13.3
Norwegian ADHD	661 cases 697 controls	33.95 ± 10.25 29.69 ± 6.52

### NCNG

The NCNG (Norwegian Cognitive NeuroGenetics) sample consists of 670 healthy individuals from whom genetic and cognitive data were collected. Participants aged 20–80 were recruited through advertisements in local newspapers in the Oslo and Bergen areas. Candidates with past or present neurological or psychiatric diseases or a history of substance abuse, learning deficits, or depression were excluded. Participants were required to be native speakers of Norwegian and to have completed basic education. Those scoring more than one standard deviation (SD) below their age norm on tests of intelligence or memory were excluded. All participants gave their informed consent for participation, which included donation of a blood sample, DNA extraction and genotyping, and storage of the remaining blood sample in a biobank. All participants read an information sheet and signed a statement of informed consent approved by the regional committee for Medical and Health Research Ethics (Southeast Norway; Project ID: S‐03116).

The cognitive tests analyzed here (summarized in Table 2) were the California Verbal Learning Test II (CVLT II) (Delis et al. 2000), the Color‐Word Interference Test (CWIT) which is part of the Delis‐Kaplan Executive Function System (DKEFS) (Delis et al. 2001), and the Vocabulary and Matrix Reasoning subtests from Wechsler Abbreviated Scale of Intelligence (WASI) (Wechsler 1999), which were used to estimate general cognitive abilities (IQ). The CVLT II scores several parameters of episodic memory function, including a total learning score, and free recall after 30 min. The CWIT comprises four conditions: the naming of color patches (1), reading of color words (2), color‐word inhibition (3), and color‐word inhibition/switching (4), with the third included in this study. For a more thorough description of the NCNG sample and tests, see (Espeseth et al. 2012).

**Table 2:** Summary of the complementary cognitive tasks analyzed in the NCNG and Betula samples

Cognitive aspect tested	NCNG test	Betula test	Measure
Semantic knowledge	Vocabulary (WASI)	Vocabulary	Word comprehension
Visuospatial ability	Matrix Reasoning (WASI)	Block design test	Visuospatial ability
Estimated IQ	Estimated IQ from Vocabulary and Matrix Reasoning (WASI)	Estimated from Vocabulary and the Block design test	Intellectual function
Episodic memory	California Verbal Learning Test‐II: Total learning	Encoding and free recall of short sentences	Verbal learning
Delayed episodic memory	California Verbal Learning Test‐II: Recall	Delayed cued recall of nouns in sentences	Verbal recall
Processing speed	Third condition of CWIT from D‐KEFS	Letter‐digit substitution	Response inhibition

WASI, Wechsler Abbreviated Scale of Intelligence; CWIT, Color‐Word Interference Test; D‐KEFS, Delis–Kaplan Executive Function System.

The whole sample underwent genome‐wide genotyping using the Illumina Human610‐Quad Beadchip. The description of quality control is provided in Espeseth et al. (2012). Imputation was performed according to the ENIGMA protocol (Stein et al. 2012) (http://enigma.loni.ucla.edu/protocols/) with the use of MACH (Li et al. 2009, 2010) and minimac (Howie et al. 2012) imputation software. The 1000 Genomes Project reference haplotype dataset, Interim Phase 1 release for the European populations (EUR) was used. SNPs with an imputation quality estimate *r* ^2^ value >0.5 were considered to be successfully imputed and the most likely genotypes were derived from the dosage values. A postimputation quality control was performed to exclude SNPs with a call rate <0.95, minor allele frequency (MAF) <0.01, and Hardy–Weinberg Equilibrium (HWE; exact test) *P*‐value <0.001. After quality control of the NCNG genotyping data, 645 individuals remained. The markers located in the *ARC* gene (+/−10 kb) or in LD were identified using the LDsnpR tool (Christoforou et al. 2012). This set consisted of two SNPs that were genotyped and 69 SNPs that were imputed.

### Betula

The Betula Project is a longitudinal study (est. 1988) on aging, memory, and dementia. This sample consists of individuals that were assessed for cognitive functions of memory, speed of processing, and attention (Nilsson et al. 2004;). The measures of cognitive function used here were selected to allow comparison with the NCNG sample (see Table 2). We used a subset of 1800 Betula samples for which DNA was available. Samples were genotyped for 19 markers in the *ARC* gene, which were selected to ensure a complete coverage of the *ARC* ±10 kb region and SNPs in LD. A special emphasis was placed on rare SNPs in the region identified by the 1000 Genomes Project (http://www.1000genomes.org/). Genotyping was done with a custom Illumina iSelect array. Samples were subjected to stringent quality control in PLINK. SNPs were excluded from the analysis if they had a failure rate <0.95, MAF <0.01, or HWE exact test *P* < 0.001. After quality control of the iSelect genotyping data, 1742 individuals remained. All participants signed informed consent, in accordance with the guidelines of the Swedish Council for Research in Humanities and Social Sciences.

### Analysis of NCNG and Betula samples

To test whether SNPs were associated with the different cognitive tests selected, we performed linear regression analyses using PLINK (Purcell et al. 2007). Age and sex were set as covariates except for the IQ measure, which had already been adjusted for age.

### Norwegian ADHD sample

After excluding known duplicates and family members, individuals with self‐reported mental retardation and samples not meeting the quality control criteria outlined below (143 samples), the Norwegian ADHD sample consists of 661 adult ADHD cases and 697 population controls. For information about recruitment, phenotyping and DNA collection, see (Johansson et al. 2008; Halmøy et al. 2009). Tag SNPs were selected to adequately cover the *ARC* gene and SNPs in LD (for detailed protocol see (Le Hellard et al. 2009). Quality control was done using PLINK (Purcell et al. 2007), and consisted of excluding individuals with <90% call rate, as well as markers with <95% genotype frequency and those significantly out of Hardy–Weinberg equilibrium (*P* = 0.01). The *ARC* gene was tagged using HapMap phase III data, spanning a region of 38.4 kb. The tagging region did not overlap with other genes. A total of nine SNPs were genotyped at the CIGENE platform at the Norwegian University of Life Sciences at Ås, using the MassARRAY iPLEX system (Sequenom, San Diego, CA). One SNP was excluded for HWE *P*‐value <0.001, leaving 8 SNPs for further analysis. Statistical analyses were done by using single‐point analysis through logistical regression in PLINK using gender as a covariate.

## Results

### ARC genetic variants and cognitive abilities

Summary statistics of the NCNG cognitive tasks are displayed in Table 3. We extracted all of the imputed and genotyped markers located within the *ARC* gene ±10 kb from the GWAS performed on the NCNG sample (Espeseth et al. 2012). We also included all the markers that were in LD with *ARC* (*r* ^2^ > 0.8) using the LDsnpR tool (Christoforou et al. 2012). Thus, all genetic variation in *ARC* and correlated with *ARC* was included. Regions in LD with *ARC* can be visualized in the LD plot of the *ARC* locus (Figure S1). Linear regression analysis revealed nominally significant association (*P* < 0.05, without multiple testing correction) between SNPs in the 20 kb region flanking *ARC* and the visuospatial ability subset, delayed episodic memory, and episodic memory (Table 4). Although the data were extracted from a genome wide association study, we here calculated a less conservative study level. We factored in the number of markers tested (71) and their nonindependence [i.e., their linkage disequilibrium (LD)], as well as the phenotype correlation. We calculated that a correct value of study‐wide significance would be *P*‐value <0.001 for association with cognitive function. Thus, none of the associations remained significant at the study‐wide level. For a complete list of each SNP's *P*‐value, see Table S1 for the NCNG sample.

**Table 3:** Summary statistics of NCNG cognitive tasks

Cognitive test	Mean	Median	95% CI
NCNG			
Vocabulary (WASI)	65.5	67	0.54
Matrix Reasoning (WASI)	27.8	29	0.35
Estimated IQ from Vocabulary and Matrix Reasoning (WASI)	118.9	120	0.80
California Verbal Learning Test‐II: Total learning	56.9	58	0.83
California Verbal Learning Test‐II: Recall	13.2	14	0.21
Third condition of CWIT from D‐KEFS	52.2	50	0.98

WASI, Wechsler Abbreviated Scale of Intelligence; CWIT, Color‐Word Interference Test; D‐KEFS, Delis–Kaplan Executive Function System.

**Table 4:** Nominal associations of *ARC* SNPs with cognitive abilities (NCNG: *N* = 670; Betula: *N *= 1800

SNP	M/m allele	MAF^a^	Semantic Knowledge *P*‐value [Sample]^b^	Visuospatial Abilities *P*‐value [Sample]^b^	Delayed Episodic Memory *P*‐value [Sample]^b^	Estimated IQ *P*‐value [Sample]^b^	Episodic Memory *P*‐value [Sample]^b^	Processing Speed *P*‐value [Sample]^b^
rs13273921	T/C	0.486	—	—	0.045 [N]	—	—	—
rs13260813	A/C	0.092	—	—	—	—	0.03 [B]	—
rs28625055	G/A	0.225	—	0.045 [B]	0.03 [B]	—	—	—
rs79905110	G/A	0.095	—	0.03 [N]	—	—	—	—
rs10105842	C/T	0.134	—	0.03 [B]	—	—	—	—
rs10110456	G/A	0.088	—	0.04 [B]	—	—	—	—
								

—, Indicates nonsignificance.

a MAF = Minor Allele Frequency (European).

b N = NCNG; B = Betula.

Analogous cognitive tests were analyzed from a sample of 1800 Betula participants (Table 2). Ten *ARC* genetic variant markers were genotyped using an iSelect platform, and a linear regression analysis was performed with age and gender as covariates. No association was detected between any *ARC* SNP and the cognitive functions tested. A summary of all nominally associated SNPs in the two samples is listed in Table 4. For a complete list of each SNP's *P*‐value in the Betula sample, see Table S2.

### ARC genetic variants and ADHD

In our Norwegian ADHD sample, we genotyped and analyzed 8 tagging SNPs of *ARC* and the surrounding region in a sample of 661 adult ADHD patients and 697 controls. Single‐point analyses did not reveal any significant association between ADHD and *ARC* genotypes. The *P*‐values of SNP analyses are given in Table S3.

## Discussion

The study presented here indicates that common genetic variants within *ARC* (±10 kb) are not associated with normal variation in human cognition in our samples or with ADHD.

**Figure 1:**
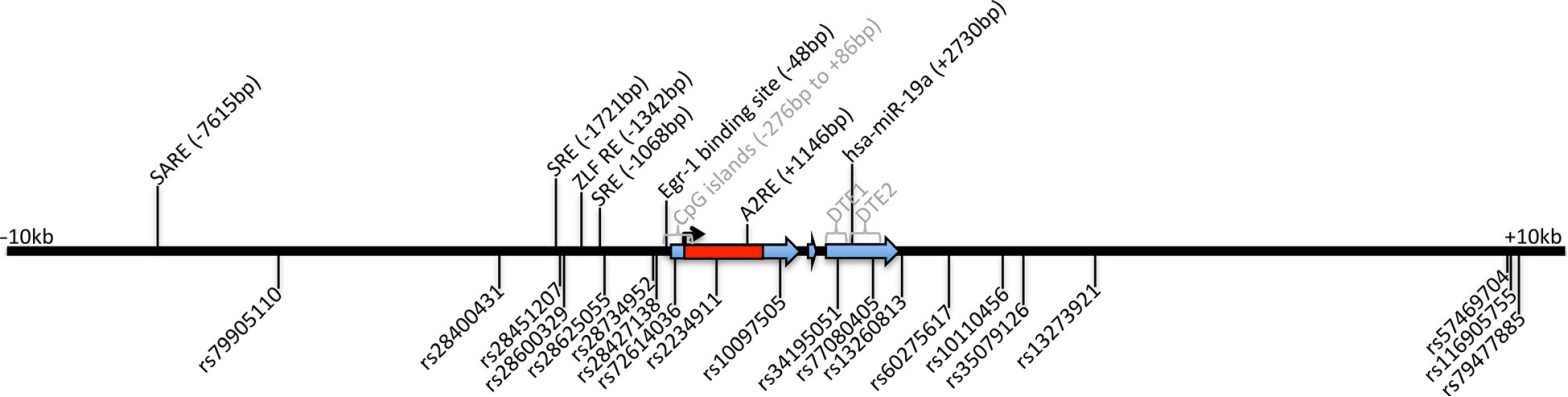
Regulatory features of *ARC* gene and location of SNPs. The red region indicates the coding sequence and the blue regions are exons. Regulatory features of *ARC* are indicated on the top while nominally associated *ARC* SNPs are indicated on the bottom. (SARE = synaptic activity‐response element; SRE = serum response element; ZLF RE = Zeste‐like factor response element; A2RE = hnRNP A2 response element; hsa‐miR‐19a = *Homo sapiens* microRNA‐19a predicted binding site; DTE = dendritic targeting element).

We have previously shown evidence for significant associations between variants in *DCLK1* and variants in *BDNF* and *ARC*, which affected verbal memory and general cognitive abilities in a smaller NCNG sample (Le Hellard et al. 2009). Here, using an extended NCNG sample and an additional sample (the Betula) as well as higher coverage of the *ARC* gene, no associations between common genetic variants within *ARC* and cognition were found. However, we cannot completely exclude association between genetic variants in *ARC* and cognitive abilities for several reasons. First, *ARC* variants might associate with specific cognitive abilities other than those tested here. Our analysis included word comprehension, visuospatial ability, intellectual function, verbal learning, verbal recall, and response inhibition, but more detailed measures qualifying as cognitive endophenotypes and tests for nonverbal episodic memory should be included in further studies. Second, recent GWAS confirm that genetic factors contribute to a large extent to the interindividual variability in cognitive abilities (Davies et al. 2011), but their individual effect is very small and difficult to detect with classical GWAS (Davies et al. 2015). The nonsignificant study‐wide associations between *ARC* SNPs and the traits examined here is consistent with the intricate, polygenic genetic architecture of complex traits such as cognition and psychiatric disorders. It is also in line with the assumption that much of the heritability that cannot yet be explained by GWAS (aka the “hidden heritability”), but that is shown to be attributable to common variants, could partially be accounted for by many variants of very small effect size (Visscher et al. 2012). Larger sample sizes may provide the power needed to detect these effects. Lastly, much is still to be learned about *ARC*. It is exquisitely regulated at nearly every level (epigenetically, mRNA transport, by miRNAs, mRNA and protein degradation, etc.), and other mechanisms are certainly yet to be described. Some known regulatory regions of *ARC* are shown in Fig. 1, along with the location of the nominally associated SNPs. Based on current knowledge of *ARC* regulatory regions, none of the nominally significant SNPs in this study are likely to have functional significance. Large‐scale sequencing is needed to identify the full range of SNPs present within *ARC,* in *ARC‐*regulating regions, and other SNPs in *ARC* signaling complexes.

Recent studies on the genetic etiology of SCZ have reported de novo copy number variants (CNVs), small de novo mutations, and rare coding variants in genes encoding for proteins implicated in an *ARC* signaling complex (Kirov et al. 2012; Fromer et al. 2014; Purcell et al. 2014). While substantial further study is required, these studies have pointed at the involvement of an *ARC* complex in SCZ. It will thus be necessary to carry out similar studies to characterize the implication of rare *ARC* variants and in genes of *ARC* complexes in cognitive functions. We also note that none of the common *ARC* variants reported in the Psychiatric Genomics Consortium database showed significant association with SCZ (Ripke et al. 2014).

Impairments in cognitive function are observed across the major neuropsychiatric disorders (Gottesman and Gould 2003; Hasler et al. 2006). We therefore utilized our Norwegian ADHD sample to test for association between *ARC* SNPs and ADHD, but found no significant associations. Since present ADHD diagnostic categories are rather heterogeneous, more precise phenotypic categories should be analyzed. More importantly, a more thorough association analysis between *ARC* variants and other neuropsychiatric disorders deserves consideration. As a tightly controlled gene central to brain plasticity and cognition, variation in *ARC* may still prove to confer beneficial and/or deleterious effects on human cognitive abilities.

## Conflict of Interest

None declared.

## Supporting information


**Figure S1.** Linkage disequilibrium (LD) and haplotype block structure of the region tested around the *ARC* gene (chromosome 8q24.3).

**Table S1**. Complete set of computed data (linear regression analysis) for NCNG Sample.
**Table S2**. Complete set of computed data (linear regression analysis) for Betula Sample.
**Table S3**. Complete set of computed data (single point analyses) for the Norwegian ADHD Sample.
