# Supplementary figures and images for: Common variants in the ARC gene are not associated with cognitive abilities
Source: Brain Behav. 2015 Sep 3;5(10):e00376. doi: 10.1002/brb3.376 (PMC4614059; doi:10.1002/brb3.376)

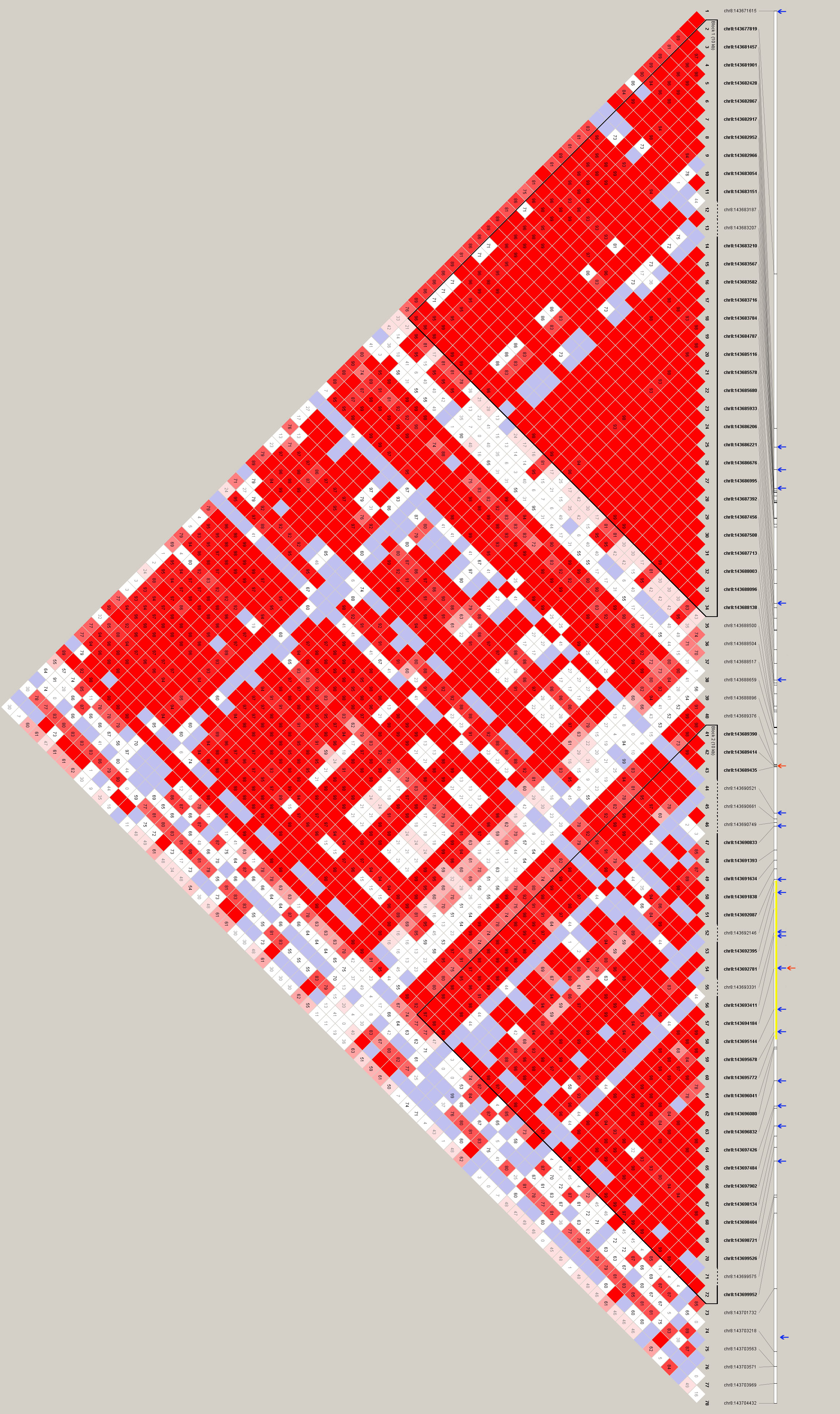

Supplement: Supplementary file 1 — Figure S1. Linkage disequilibrium (LD) and haplotype block structure of the region tested around the ARC gene (chromosome 8q24.3). [file BRB3-5-0h-s001.jpg]
